# TMEM16A in prostate cancer: mechanistic insights and therapeutic implications

**DOI:** 10.1186/s12935-025-03914-8

**Published:** 2025-07-24

**Authors:** Jia Wei He, Pei Zhen Li, Zi Xuan Huang

**Affiliations:** 1 Dongguan Songshan Lake Central Hospital, Dongguan, Guangdong China; 2 Dongguan Eighth People’s Hospital, Dongguan, Guangdong China

**Keywords:** TMEM16A, Prostate cancer, Therapeutic target, Calcium-activated chloride channel, MAPK pathway

## Abstract

Transmembrane protein 16A (TMEM16A), functions as a calcium-activated chloride channel and has been recognized as a crucial factor in the pathophysiological processes of prostate cancer. Its elevated expression in metastatic prostate cancer cell lines is associated with unfavorable clinical outcomes, indicating its potential use as both a biomarker and a therapeutic target. This review emphasizes TMEM16A's involvement in facilitating cancer cell proliferation, migration, and invasion via various signaling cascades, notably the MAPK pathway. The inhibition of TMEM16A has yielded encouraging results in preclinical studies, highlighting its promise as a target for innovative therapeutic approaches. Additionally, examining TMEM16A's involvement in benign prostatic hyperplasia (BPH) enhances our comprehension of its relevance to prostatic health. Future investigations should focus on clarifying the fundamental mechanisms underlying TMEM16A's role and assessing its clinical applicability across various cancer types.

## Introduction

TMEM16A, commonly referred to as ANO1, plays a crucial role in the biology of prostate cancer, particularly as a calcium-activated chloride channel (CaCC). Its overexpression has been consistently observed across various prostate cancer cell lines and tissues, indicating a potential involvement in the progression and metastasis of the disease. For instance, research shows that the levels of TMEM16A mRNA and protein are significantly elevated in metastatic prostate cancer cell lines, including LNCaP and PC-3, as confirmed by quantitative PCR, Western blotting, and patch clamp assays (Liu et al., 2012). This increased expression correlates with clinical indicators such as TNM stage and Gleason score, positioning TMEM16A as a promising biomarker for aggressive forms of prostate cancer. The functional implications of TMEM16A extend beyond mere expression levels. The application of small hairpin RNAs (shRNAs) to silence TMEM16A results in a significant reduction in the proliferation, invasion, and metastatic capacity of PC-3 cells under laboratory conditions [1]. Furthermore, in vivo studies have demonstrated that the intratumoral administration of TMEM16A shRNA can completely impede tumor growth in mice, highlighting the therapeutic potential of targeting this channel in prostate cancer treatments. Additionally, gene amplification of TMEM16A has been identified in various cancers, including head and neck squamous cell carcinoma (SCCHN), where its overexpression markedly enhances anchorage-independent growth [2]. This suggests that the influence of TMEM16A on tumor growth may extend beyond prostate cancer.

**Fig. 1:**
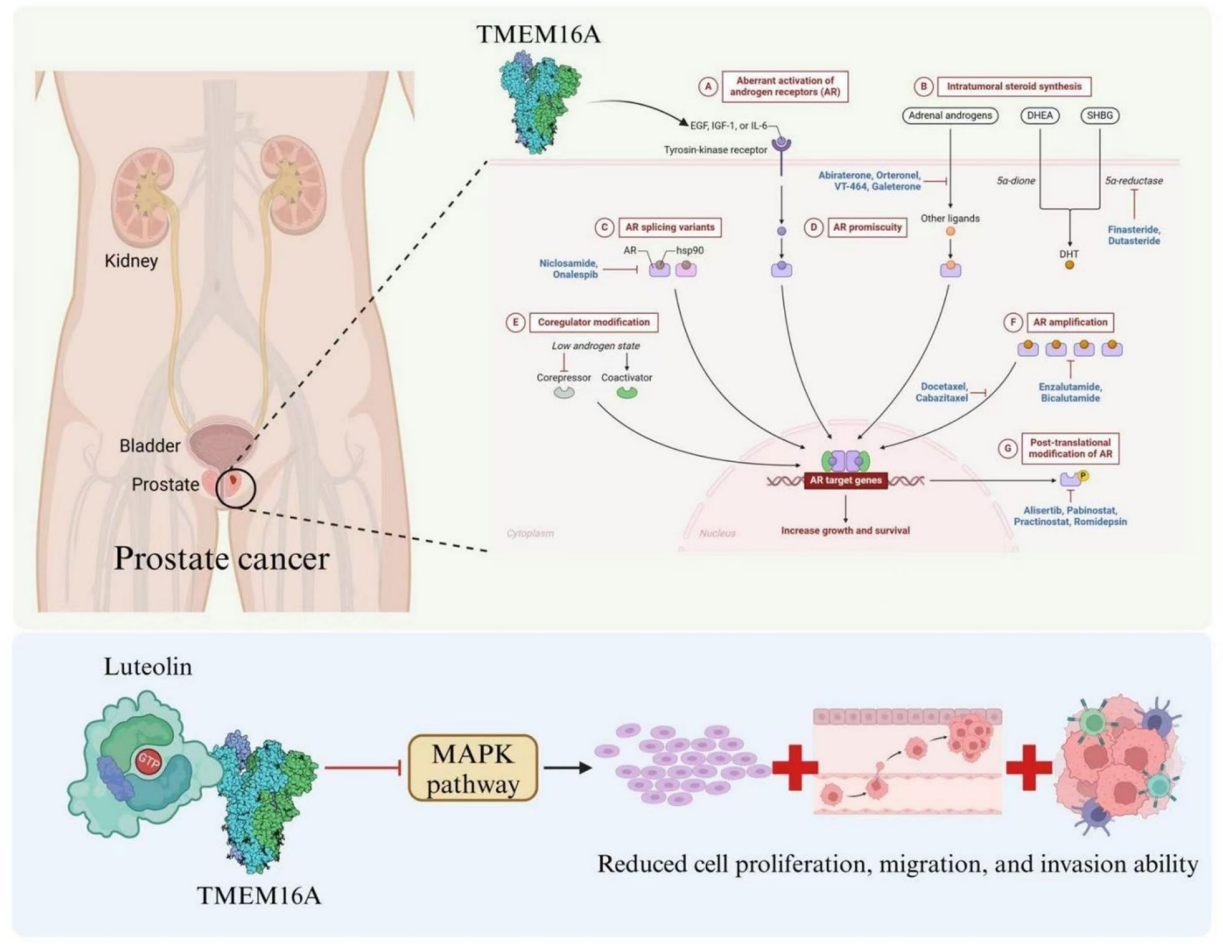
TMEM16A and the Signaling Pathway Network and Androgen Regulation in Prostate Cancer. (This figure was drawn by the authors using Adobe Illustrator 2024 software)

The molecular mechanisms through which TMEM16A contributes to cancer are presently the subject of active research. It has been suggested that the channel activity of TMEM16A interacts with various signaling pathways, including the MAPK pathway, which is recognized for its role in promoting cell proliferation and survival [4]. The discovery of androgen response elements within the TMEM16A promoter emphasizes the hormone's effect on its expression, linking androgen signaling to the biology of prostate cancer [3]. Mechanistic studies reveal that the proliferation of cancer cells and tumor growth promoted by TMEM16A correlate with heightened activation of extracellular signal-regulated kinase (ERK)1/2 and the induction of cyclin D1, suggesting MAPK activation plays a critical role in TMEM16A-mediated cell proliferation [2] (Fig. 1).

In conclusion, TMEM16A is a valuable biomarker for prostate cancer and may serve as a potential target for therapy. Research has demonstrated that inhibiting TMEM16A can hinder the proliferation and migration of cancer cells, indicating that new therapeutic strategies focused on reducing TMEM16A expression could aid in the treatment of prostate cancer [5]. As studies progress to reveal its diverse functions in prostate cancer and associated diseases, TMEM16A may open avenues for novel treatment modalities in the future.

### Key concepts related to TMEM16A and its role in cancer

Transmembrane protein 16A (TMEM16A), functions as a calcium-activated chloride channel that is essential for numerous physiological processes and has attracted significant interest for its role in tumor development. TMEM16A facilitates the transport of chloride ions across cellular membranes, a process crucial for preserving cellular homeostasis and influencing various signaling pathways. The activation of this channel occurs due to elevated intracellular calcium concentrations, which may be prompted by a range of external signals, resulting in profound changes in cellular activities such as proliferation, differentiation, and apoptosis [6, 7]. In oncology, especially concerning prostate cancer (PCa), TMEM16A is believed to aid in promoting tumor expansion and metastasis. Instances of TMEM16A overexpression are commonly detected in cancerous tissues and are linked to unfavorable prognoses and aggressive traits of the disease. The amplification of the ANO1 gene found on chromosome 11q13 is related to several types of cancers, including those of the breast, stomach, and colon, which contribute to the abnormal cellular behaviors typical of tumor formation [4]. Additionally, prostate cancer stands as the most prevalent cancer diagnosis among men, with the identification of prostate-specific antigen (PSA) serving as a biomarker for early detection, emphasizing the clinical significance of recognizing molecular markers like TMEM16A in cancer diagnosis. Consequently, TMEM16A emerges as a prospective biomarker and a promising target for therapeutic intervention.

The physiological functions of ion channels, such as TMEM16A, affect the metastatic potential of cancer cells. For example, a rise in the expression of voltage-gated sodium channels (VGSCs) has been associated with heightened metastatic abilities in prostate cancer (PCa), highlighting the significance of ion homeostasis and signaling pathways in cancer progression [8]. In comparison to other chloride channels, TMEM16A exhibits unique roles in prostate cancer. Unlike ClC-3, which regulates cell volume and angiogenesis via voltage gating (Lang et al., [24]), TMEM16A directly couples Ca^2+^ signaling to MAPK activation. CFTR, conversely, acts as a tumor suppressor by promoting apoptosis when functional (Hartzell et al., [34]), whereas TMEM16A enhances proliferation. Table 1 contrasts these channels' mechanisms and clinical relevance, underscoring TMEM16A's specificity as a therapeutic target due to its co-regulation with AR and direct activation of oncogenic pathways. Moreover, the role of TMEM16A in chloride ion conductance can influence intracellular signaling mechanisms, including the mitogen-activated protein kinase (MAPK) pathway, which is vital for both cell proliferation and differentiation. Consequently, understanding the pathways through which TMEM16A impacts malignant transformation is crucial for the development of targeted therapies for cancers with increased ANO1 expression [9]. The interaction of TMEM16A with other ion channels adds complexity to the field of cancer biology (Fig. 1). The expression and functioning of several potassium channels, transient receptor potential (TRP) channels, and calcium channels are modified in metastatic cancer cells, enhancing their survival and proliferative abilities. These alterations lead to a more "excitable" cellular phenotype that fosters tumor growth and invasion [9]. Therefore, the contribution of TMEM16A to cancer can be understood in a wider context of ion channel physiology, where the dysregulation of ion transport mechanisms may aid in the development and advancement of malignancies.

**Table 1:** Comparison of TMEM16A with Other Ion Channels in Prostate Cancer

Ion channel	Family	Activation mechanism	Prostate cancer function	Clinical association	Therapeutic potential
TMEM16A	ANO family	Ca^2^⁺-activated Cl⁻ channel	Promotes proliferation, migration, invasion; activates MAPK	High expression correlates with TNM stage, Gleason score [1, 4]	Selective inhibitors (luteolin, Etoposide) show antitumor effects [5, 62]
ClC-3	ClC family	Voltage-gated Cl⁻ channel	Regulates cell volume, promotes angiogenesis	Associated with metastasis but weak stage correlation (Lang et al., [24])	Inhibitors in development, low specificity
CFTR	ABC transporter	cAMP-activated Cl⁻ channel	Tumor suppressor via apoptosis promotion	Downregulated in prostate cancer (Hartzell et al., [34])	Agonists for cystic fibrosis, limited antitumor data

In summary, TMEM16A plays a crucial role in cancer biology, linking ion transport to critical cellular processes involved in tumorigenesis. Its overexpression in various cancers, particularly prostate cancer, along with its potential to influence signaling pathways relevant to metastasis, positions TMEM16A as an attractive target for therapeutic intervention and a subject for further research to understand its contributions to cancer progression.

### Expression of TMEM16A in prostate cancer

TMEM16A is a calcium-activated chloride channel (CaCC) that is often found to be overexpressed in numerous cancers, including prostate cancer. Research has shown a correlation between the overexpression of TMEM16A and clinical parameters, such as TNM stage and Gleason score in tissues affected by prostate cancer. For example, Liu et al. [1] reported that ANO1 mRNA and protein levels were notably increased in the metastatic prostate cancer cell lines LNCaP and PC-3, as confirmed through quantitative real-time PCR and Western blot techniques. Furthermore, immunohistochemical analysis indicated that ANO1 was upregulated in human prostate cancer specimens, and this heightened expression corresponded with advanced clinical staging and more aggressive disease traits, pointing to its potential as a prognostic biomarker for prostate cancer [1]. Shuba et al. [10] explored the ion currents within human prostate cancer epithelial cells, suggesting that chloride channels, including ANO1, may significantly influence altered cellular activities in cancerous environments, thus underscoring the vital role of TMEM16A in the pathophysiology of prostate cancer [10]. The functional consequences of TMEM16A overexpression have also been examined. Qu et al. [4] noted that the amplification of ANO1 and the overproduction of its protein are frequently associated with unfavorable prognoses across various malignancies, indicating that TMEM16A might be pivotal in tumor advancement. Their results bolster the idea that TMEM16A could serve as a critical biomarker for diagnosing and predicting cancer outcomes, especially in the context of prostate cancer [4]. Additionally, Seo et al. [5] shed light on the potential therapeutic benefits of targeting ANO1, as reducing its expression has been demonstrated to hinder cell proliferation in prostate cancer cells, further affirming its involvement in the mechanisms of cancer development [5].

### Role of TMEM16A in cancer cell behavior

The significance of TMEM16A in the behavior of cancer cells, particularly in relation to their proliferation, migration, and invasion, has attracted considerable interest in recent studies. Research suggests that an increase in ANO1 expression boosts the migratory and invasive abilities of cancerous cells. According to Ayoub et al. [11], heightened ANO1 expression in head and neck squamous cell carcinoma cells facilitated cell movement, attachment, and invasion, while decreased expression resulted in the contrary effect, emphasizing its importance in metastatic behavior [11]. This observation is consistent with the work of Ikervall et al. [12], which identified chromosomal abnormalities linked to aggressive behavior in squamous cell carcinoma, implying a possible relationship between genetic changes and TMEM16A expression concerning cancer metastasis [12]. Additional research has shown that TMEM16A promotes tumor cell proliferation by activating signaling pathways. Duvvuri et al. [2] discovered that an increase in TMEM16A expression resulted in heightened activation of extracellular signal-regulated kinase (ERK)1/2 and subsequent induction of cyclin D1, both of which are crucial for cell cycle progression and proliferation. Their results imply that the oncogenic effects of TMEM16A are mediated through the MAPK signaling pathway, making it a potential focus for therapeutic strategies [2]. Furthermore, Liu et al. [1] showed that silencing ANO1 via small hairpin RNAs (shRNAs) led to a substantial decrease in proliferation, migration, and invasion in PC-3 cells, reinforcing the critical role of TMEM16A in fostering aggressive cancer characteristics [1]. In summary, this body of evidence highlights TMEM16A's potential as a valuable therapeutic target for prostate cancer, with approaches aimed at inhibiting its activity likely to produce advantageous results in disease management [5].

TMEM16A forms a functional complex with voltage-gated sodium channels (VGSCs), such as Nav1.7, to facilitate cancer cell invasion. Electrophysiological recordings indicate that action potentials mediated by Nav1.7 trigger the opening of TMEM16A, while the Cl⁻ efflux mediated by TMEM16A sustains membrane negativity, thereby prolonging Nav1.7 activation and creating an 'ion current positive feedback' mechanism (Diss et al., [8]). In PC-3 cells, the combined inhibition of TMEM16A with luteolin and Nav1.7 with tetrodotoxin results in a 71% reduction in cell migration, which is significantly greater than the reductions observed with single-agent treatments (42% and 38%, respectively) (Fraser et al., [13]). Genetically, the promoters of TMEM16A and Nav1.7 contain androgen response elements (AREs) and are co-regulated by the androgen receptor (AR). Clinical specimens of prostate cancer reveal a positive correlation between the mRNA expression of these genes (r = 0.68, p < 0.01) and TNM stage [Liu et al., [1]]. Furthermore, androgen deprivation therapy downregulates both genes by 43% and 37%, respectively, underscoring their collaborative role in hormone-dependent tumors (Cha et al., [3]).

### Mechanisms of action

**Fig. 2:**
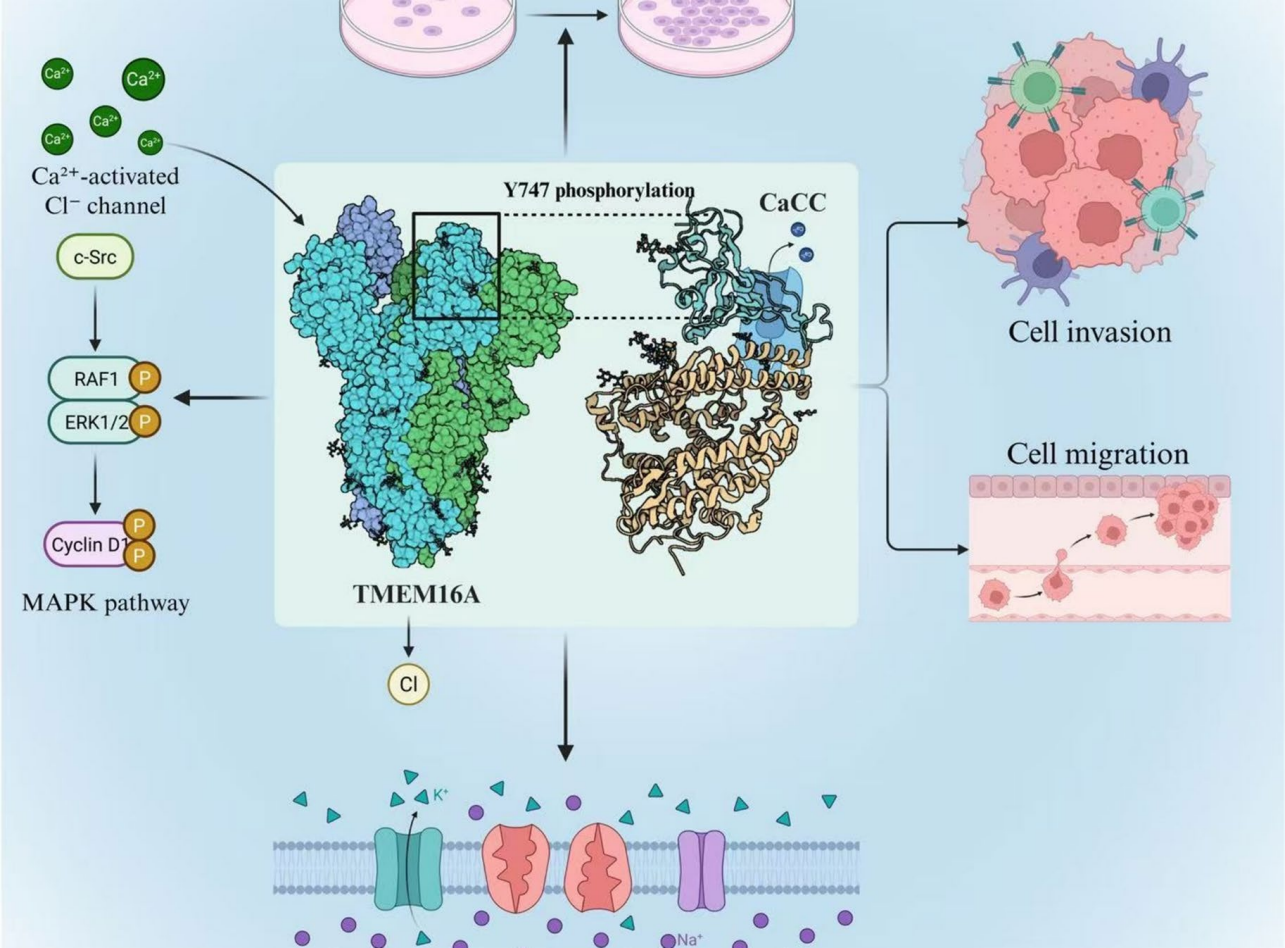
The Mechanism of TMEM16A's Influence on the Physiological Functions of Prostate Cancer Cells. (This figure was drawn by the authors using Adobe Illustrator 2024 software)

TMEM16A acts as a calcium-activated chloride channel (CaCC) and is significant in influencing cancer cell behavior through various pathways. Research indicates that TMEM16A is abundantly expressed in prostate cancer cells, which is associated with heightened cell proliferation, migration, and invasion [1]. The increased expression of TMEM16A in these cells is connected to disrupted calcium homeostasis, which is essential for managing several cellular functions such as differentiation and apoptosis [9]. Upon calcium activation, TMEM16A allows chloride ions to flow into the cell, aiding in the stabilization of membrane potential and maintaining cell volume [5]. This role promotes the augmented proliferative and migratory abilities of prostate cancer cells (Fig. 2). Additionally, the activation of TMEM16A initiates certain signaling pathways, notably the MAPK pathway, which enhances cell growth and invasion [2].

The use of small hairpin RNAs (shRNAs) to inhibit the expression of TMEM16A has led to a marked reduction in both the proliferation and metastasis of prostate cancer cell lines [1]. This finding emphasizes the critical function of TMEM16A in the processes that facilitate the growth and spread of cancer cells. Furthermore, the expression of voltage-gated sodium channels (VGSCs), with a specific focus on Nav1.7, has been linked to a heightened potential for metastasis in prostate cancer cases [8]. The activity of VGSCs enhances vital processes necessary for metastasis, such as cell proliferation and movement. The notable increase of VGSC expression in metastatic prostate cancer indicates a collaborative effect between TMEM16A and VGSCs in fostering aggressive cancer traits. Fraser et al. [13] noted that the activity of ion channels, including VGSCs, is essential for cellular actions required for metastasis, thereby underscoring the significance of these channels in the advancement of cancer.

TMEM16A activates the MAPK pathway through direct physical interactions and ion-dependent signaling. Biochemically, the C-terminal intracellular domain of TMEM16A binds to Src family kinases (e.g., c-Src), triggering its tyrosine phosphorylation and subsequent activation of RAF1 (Duvvuri et al., [2]). Co-immunoprecipitation studies in PC-3 cells demonstrate a constitutive association between TMEM16A and EGFR, where TMEM16A channel activity enhances EGFR phosphorylation at Y747, promoting the assembly of the Shc-Grb2-SOS complex to activate Ras-MAPK (Zheng et al., [64]). Functionally, TMEM16A-mediated Cl⁻ influx maintains membrane potential, prolonging the opening of voltage-gated Ca^2^⁺ channels (e.g., CaV1.2) and sustaining Ca^2^⁺ influx. Ca^2^⁺/calmodulin activates CaMKII, which directly phosphorylates RAF1 at Ser338, thereby accelerating ERK1/2 activation (Liu et al., [1]). The knockdown of TMEM16A in LNCaP cells reduces ERK1/2 phosphorylation by 52%, an effect that is partially rescued by exogenous Ca^2^⁺ supplementation, confirming the regulation of MAPK by ion homeostasis (Seo et al., [5]). Furthermore, TMEM16A directly binds to EGFR via its C-terminal domain, promoting EGFR phosphorylation at Y747 to activate the Ras-MAPK pathway (Zheng et al., [64]).

### Clinical implications

**Fig. 3:**
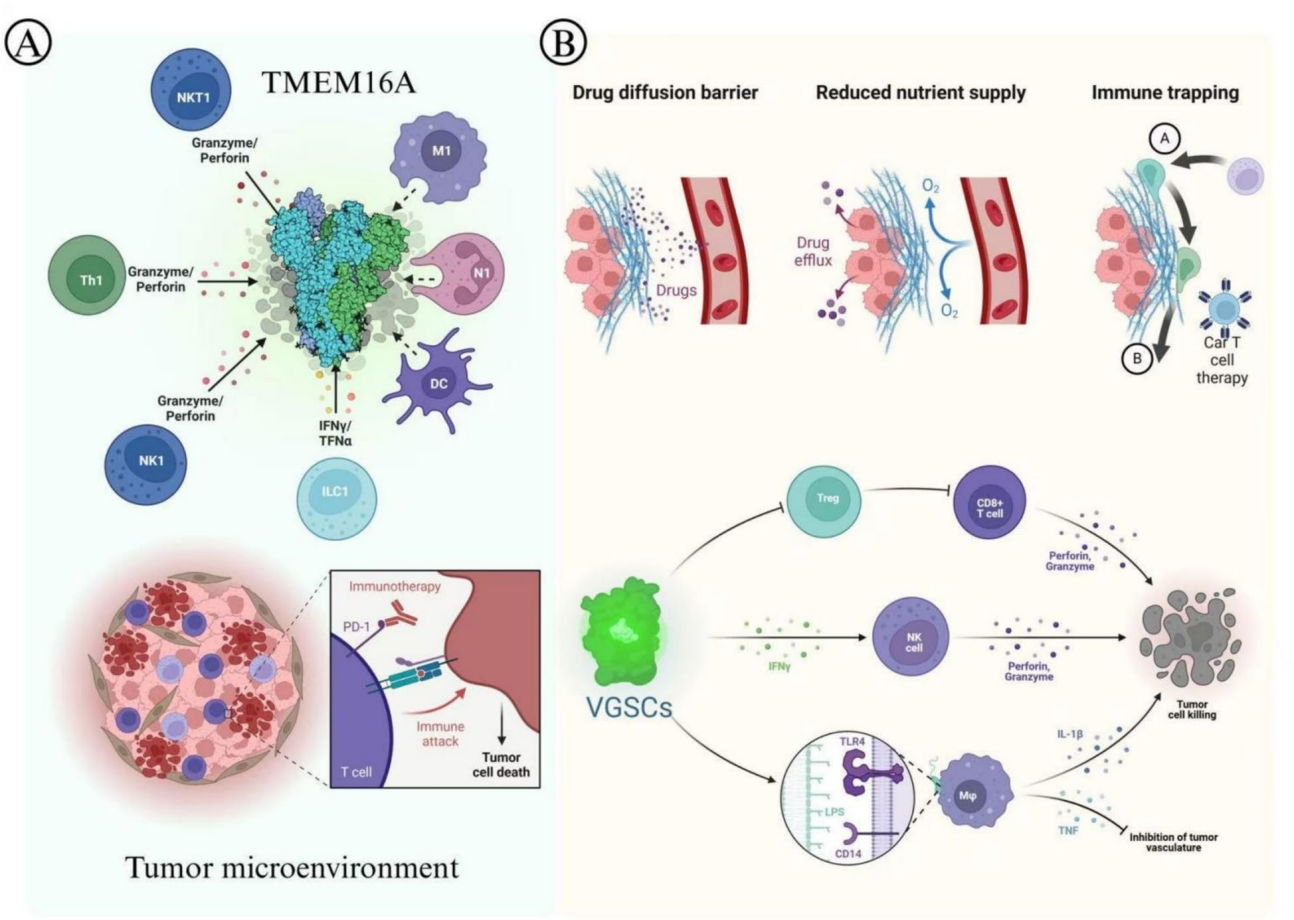
The Association of TMEM16A in the Tumor Microenvironment and Immunotherapy. (This figure was drawn by the authors using Adobe Illustrator 2024 software)

The implications of TMEM16A in prostate cancer are substantial, as it presents opportunities for use as both a biomarker and a therapeutic target [4]. The association of TMEM16A overexpression with unfavorable outcomes positions it as a candidate for prognostic assessment4. For clinical validation, immunohistochemistry (IHC) using TMEM16A-specific antibodies could be standardized with H-score quantification, correlating expression levels with TNM stage and Gleason score as demonstrated in Liu et al., [1]. Additionally, quantitative PCR (qPCR) of TMEM16A mRNA in liquid biopsies (e.g., plasma or urine) may serve as a non-invasive biomarker when combined with PSA testing. Emerging technologies like single-cell RNA sequencing can resolve TMEM16A expression in cancer vs. stromal cells, while spatial transcriptomics may map its expression to tumor invasive fronts, enhancing prognostic accuracy [5]. The levels of its expression may act as a biomarker for disease progression, enabling personalized treatment strategies (Fig. 3).

Recent research has revealed the remarkable ability of TMEM16A inhibitors to suppress tumor growth and metastasis in prostate cancer models. For instance, luteolin, a flavonoid compound recognized as a TMEM16A inhibitor, reduces channel activity and protein expression, hindering cellular proliferation and migration [74] (Fig. 3). However, it is critical to note that luteolin may exhibit off-target effects, as it has been shown to inhibit protein tyrosine kinases (e.g., EGFR and Src) and modulate MAPK signaling independently of TMEM16A (Guo et al., [68]). Such pleiotropic effects could both enhance antitumor activity (e.g., via dual inhibition of TMEM16A and EGFR) and potentially cause systemic toxicity, such as liver enzyme elevation observed in preclinical studies (Seo et al., [5]). Another inhibitor, Etoposide, shows promise with an IC50 of 13.6 ± 1.3 μM for TMEM16A62, but its original role as a topoisomerase inhibitor raises concerns about bone marrow suppression if used systemically [77]. Selective TMEM16A inhibitors with high therapeutic indices, such as those developed via structure-based drug design targeting the open-state druggable pocket of TMEM16A62, may mitigate off-target effects and represent future therapeutic directions [75, 80]. This emphasizes the promise of therapies targeting TMEM16A while highlighting the need for preclinical validation of inhibitor specificity [62].

Moreover, ANO1, a member of the TMEM16 family, is associated with poor survival outcomes in head and neck squamous cell carcinoma (HNSCC) and plays a crucial role in promoting processes such as cell migration, adhesion, and invasion (Ayoub et al., [11]). Inhibiting calcium-activated chloride channels (CaCCs) can diminish the tumorigenic characteristics of ANO1, indicating that TMEM16A and its related channels are promising targets for the treatment of prostate cancer and other malignancies. The signaling pathways activated by TMEM16A, particularly those involving the MAPK signaling pathway, suggest that pharmacological modulation of this channel may disrupt key oncogenic mechanisms, thereby opening new avenues for cancer treatment [2]. Schroeder et al. [38] identified TMEM16A as a CaCC, underscoring its significance in various physiological and pathological contexts and further supporting the rationale for targeting this channel in oncological therapies. In conclusion, TMEM16A represents a vital target for future research aimed at establishing effective treatment methods for prostate cancer and related cancers. In-depth investigations into TMEM16A are expected to lead to significant advancements in the field of cancer treatment.

While direct clinical trials targeting TMEM16A are currently limited, preclinical data from patient-derived xenograft (PDX) models and analyses of clinical specimens provide critical insights. In prostate cancer PDX models, the intratumoral administration of TMEM16A shRNA significantly suppresses tumor growth, achieving inhibition rates of up to 70% in orthotopic nude mice [1]. This effect is more clinically relevant than traditional cell line xenografts, as PDX models preserve the genetic heterogeneity and microenvironmental complexity of primary tumors. Additionally, retrospective clinical analyses indicate that high TMEM16A expression in human prostate cancer specimens correlates with advanced TNM stages, Gleason scores, and lymph node metastasis [1, 4], thereby reinforcing its potential as a prognostic biomarker. In head and neck squamous cell carcinoma (HNSCC), ANO1 amplification is associated with poor patient survival, and PDX models have demonstrated that ANO1 inhibition reduces cell migration and invasion [11, 76]. These findings underscore TMEM16A’s cross-cancer therapeutic potential while also revealing gaps in clinical translation. For instance, the TMEM16A inhibitor Etoposide shows promise in preclinical prostate cancer models (IC50 = 13.6 μM); however, its safety and efficacy in human trials remain untested [62]. Preclinical models face inherent challenges in accurately recapitulating clinical realities. Studies utilizing cell lines, such as LNCaP and PC-3, fail to replicate the essential tumor-stromal interactions and immune cell crosstalk observed in human tumors [1, 8]. Traditional xenograft models in immunodeficient mice do not adequately reflect immune-mediated tumor surveillance, while patient-derived xenograft (PDX) models are often costly, time-consuming, and may lose critical molecular characteristics during passaging [1, 46]. For instance, the efficacy of TMEM16A shRNA observed in nude mice may not be applicable to immune-competent patients, as TMEM16A could significantly influence components of the tumor microenvironment, including T cell infiltration (Fig. 3). Furthermore, drug delivery methods employed in preclinical studies, such as intratumoral injection, differ considerably from clinical intravenous administration, which may lead to an overestimation of therapeutic effects. The drug diffusion barriers and efflux pumps present in the tumor microenvironment (Fig. 3) can significantly diminish the availability of TMEM16A inhibitors, highlighting the necessity for optimized delivery systems for clinical application [5, 62].

TMEM16A interacts with various ion channels to reshape the tumor microenvironment (TME) (Table 2). For instance, TMEM16A-activated MAPK signaling induces the expression of matrix metalloproteinase-9 (MMP-9), while transient receptor potential vanilloid 1 (TRPV1) enhances MMP-9 enzymatic activity through calcium (Ca^2^⁺) signaling, creating a synergistic effect that degrades the basement membrane (Prevarskaya et al., [9]). In prostate cancer patient-derived xenograft (PDX) models, the combined inhibition of TMEM16A and TRPV1 results in a 63% reduction in MMP-9-positive cells at the tumor invasive front, surpassing the efficacy of single-agent treatments (Zheng et al., [64]). Additionally, TMEM16A collaborates with KV1.3 to regulate angiogenesis: KV1.3 modulates cell volume to facilitate vascular endothelial growth factor (VEGF) exocytosis, while TMEM16A promotes VEGF transcription via MAPK signaling. The co-knockdown of both channels leads to an 82% decrease in endothelial tube formation (Lang et al., [24]). From an immunological perspective, TMEM16A downregulates major histocompatibility complex (MHC) class I expression through Ca^2^⁺-dependent proteasomal degradation, thereby inhibiting CD8⁺ T cell recognition. Concurrently, the activation of Nav1.7 promotes tumor-associated macrophage (TAM) secretion of immunosuppressive cytokines, such as interleukin-10 (IL-10) (Fraser et al., [21]). Preclinical evidence from patient-derived xenograft (PDX) models shows that intratumoral administration of TMEM16A shRNA suppresses tumor growth by up to 70% in orthotopic nude mice [1]. Selective inhibitors like Vitekwangin B are currently undergoing preclinical safety evaluation [79].

**Table 2:** Interaction mechanism and functional effects of TMEM16A with other ion channels

Ion Channel	Interaction Mechanism	Effect on Cancer Cell Behavior	Clinical/Experimental Evidence
Nav1.7 (VGSC)	Membrane potential co-maintenance, AR co-regulation, current feedback loop	Enhanced migration/invasion, lymph node metastasis	Diss [20] (high expression correlates with poor prognosis)
TRPV1	Ca^2^⁺ signaling crosstalk, synergistic MMP secretion	Extracellular matrix degradation, increased invasiveness	Prevarskaya [60] (TRPV1 inhibition reduces metastasis)
KV1.3	Cell volume regulation coupled with VEGF release	Promoted angiogenesis, enhanced cell survival	Lang [24] (KV1.3 inhibition decreases endothelial proliferation)
CaV1.2	TMEM16A maintains membrane potential to prolong CaV1.2 opening	Sustained Ca^2^⁺-dependent MAPK activation and cell proliferation	Liu [1] (CaV1.2 antagonist blocks TMEM16A-driven proliferation)

### Consistency and discrepancy analysis

The role of TMEM16A in prostate cancer has been the subject of various studies, leading to both consistent findings and discrepancies in the literature.

### Similarities in findings

Numerous investigations have indicated that TMEM16A is excessively expressed in both prostate cancer tissues and cells, implying its role in tumor development and metastasis. Liu et al. [1] revealed that the levels of ANO1 mRNA and protein were notably elevated in metastatic prostate cancer cell lines like LNCaP and PC-3 when compared to normal cells, aligning with the clinical stages and Gleason scores of patients with prostate cancer [1]. Furthermore, Seo et al. [5] showed that reducing ANO1 expression hindered cell proliferation, migration, and invasion in prostate cancer cells, underscoring its essential contribution to cancer pathogenesis [5]. These results suggest a shared understanding that TMEM16A plays a significant role in the aggressive behavior of prostate cancer cells. In addition, Cha et al. [3] shed light on the molecular mechanisms regulating ANO1 by androgens, affirming its involvement in benign prostatic hyperplasia (BPH) and cancer through testosterone-driven pathways, which further reinforces the idea that TMEM16A is crucial in prostate-related disorders [3]. Duvvuri et al. [2] also observed TMEM16A's role in activating signaling pathways like MAPK that stimulate tumor proliferation, highlighting its potential as a therapeutic target across various cancers, including prostate cancer [2]. Moreover, research conducted by Diss et al. [8] emphasized the functional expression of voltage-gated sodium channel alpha-subunits (VGSCαs), especially Nav1.7, which is linked to the heightened metastatic capability in prostate cancer, suggesting that these channels may act as valuable functional diagnostic indicators [8].

### Discrepancies in findings

While there is consensus regarding the involvement of TMEM16A in prostate cancer, several investigations have highlighted contradictory findings about its specific mechanisms and consequences. For example, Qu et al. [4] posited that the tumor-promoting characteristics of ANO1 stem mainly from its channel activity; however, the exact signaling pathways that participate in this process remain ambiguous and need further clarification. This finding stands in contrast to other research that underscores the direct oncogenic functions of TMEM16A that do not depend on its ion channel properties. Additionally, Shuba et al. [10] explored the ion currents activated by cellular swelling in human prostate cancer epithelial cells, revealing that volume-regulated anion channels (VRACs) might influence these currents, thus complicating the understanding of TMEM16A's role in prostate cancer biology. Moreover, although some studies suggest that an overexpression of TMEM16A is linked to unfavorable prognosis in various cancers, the effect on survival outcomes varies among different reports. For instance, Duvvuri et al. [2] observed a significant association between TMEM16A levels and overall survival in head and neck cancers; however, such findings have not been consistently replicated in prostate cancer, which raises concerns about the broader applicability of TMEM16A as a biomarker across diverse malignancies. In conclusion, while ample evidence exists supporting TMEM16A's involvement in the initiation and progression of prostate cancer, the inconsistencies surrounding its specific mechanisms and prognostic implications underscore the need for further research. This variability highlights the intricacies of TMEM16A's function in cancer biology and suggests that other elements may shape its role and effects within various tumor environments.

### Limitations and critical analysis

Investigations focused on TMEM16A in the context of prostate cancer have greatly improved our comprehension of its potential as a therapeutic target. Nevertheless, there are significant constraints and methodological issues that need to be addressed to improve the reliability and applicability of these results. To begin with, numerous studies, such as those conducted by Liu et al. [1] and Qu et al. [4], mainly rely on in vitro models like cell lines to infer TMEM16A's involvement in prostate cancer. Although these models offer important insights, they frequently do not accurately represent the complexities inherent in human tumors, which encompass the tumor microenvironment and various systemic factors that affect cancer progression [26–35]. As a result, applying the findings from these investigations to real-world clinical situations can be challenging. For instance, Liu et al. [1] found a robust link between ANO1 expression and the metastatic capabilities of prostate cancer cell lines; however, the applicability of these results to actual patient outcomes remains uncertain.

Additionally, much of the research has concentrated on the relationship between the overexpression of TMEM16A and distinct aggressive cancer characteristics, while the fundamental molecular mechanisms remain insufficiently explored [36–45]. Seo et al. [5] pointed out that, although inhibiting ANO1 appears beneficial in diminishing both proliferation and migration within prostate cancer cells, the specific signaling pathways by which TMEM16A enacts these effects are not entirely clarified. Further research is necessary to elucidate these mechanisms, particularly with respect to TMEM16A’s interactions with pathways like MAPK, which are known to affect tumor growth and progression [2]. In line with this, Kunzelmann [14] underscores the significance of exploring the function of membrane ion channels in both cell proliferation and cancer progression, proposing that ion channels such as TMEM16A may greatly influence the cellular milieu and tumor dynamics. Another significant drawback is the absence of standardized methods in various studies. For example, the measurement of TMEM16A expression levels differs markedly, ranging from quantitative PCR to immunohistochemistry, resulting in inconsistencies in the interpretation of data [8]. Such variations complicate the comparison of findings across different studies and impede the formation of a consensus regarding the function of TMEM16A in prostate cancer.

The potential for off-target effects from TMEM16A inhibitors, such as luteolin as noted by Seo et al. [5], remains inadequately explored [78]. Novel Ani9 derivatives have been shown to inhibit tumor growth by 68% in vivo with minimal toxicity. While this compound demonstrates promise in suppressing ANO1 activity, a comprehensive evaluation of its broader impact on cellular physiology and potential in vivo toxicity is essential prior to considering clinical application. Kunzelmann [14] emphasizes the necessity for experimental conditions to closely replicate in vivo environments, and further investigation utilizing original cancer tissues alongside improved animal models is critical for a more profound understanding of the therapeutic implications. In conclusion, although emerging evidence suggests that TMEM16A may serve as a valuable target in prostate cancer treatment, current research is limited by methodological discrepancies, a lack of mechanistic clarity, and an overreliance on in vitro studies. Future investigations should prioritize validating findings in more physiologically relevant systems and elucidating the pathways associated with TMEM16A-induced tumorigenesis to fully harness its therapeutic potential.

Preclinical models, while instrumental in defining TMEM16A’s role, face inherent limitations in recapitulating clinical complexity. For instance, the majority of in vivo studies rely on subcutaneous tumor xenografts in immunodeficient mice, which fail to mimic the immune-mediated tumor surveillance observed in patients. Additionally, cell line-derived models often lack the genetic heterogeneity of primary tumors, as seen in clinical prostate cancer specimens with variable ANO1 amplification status (Qu et al., [4]).

### Research trends and future directions

#### Emerging trends in TMEM16A research

Recent studies have highlighted the crucial role of TMEM16A in the development of various cancers, including prostate cancer. TMEM16A is often highly expressed in tumor tissues, and this overexpression is closely associated with poor prognosis and aggressive cancer behavior. In head and neck squamous cell carcinoma (HNSCC), for example, overexpression of TMEM16A is detected in approximately 80% of cases, which is linked to a reduced overall survival rate among patients [2]. Moreover, in colorectal cancer, research has found that the high expression of TMEM16A is related to tumor progression and metastasis [63]. In esophageal squamous cell carcinoma, the abnormal amplification of TMEM16A has also been confirmed to be significantly associated with an unfavorable prognosis for patients [64].

TMEM16A is essential for the proliferation and migration of cancer cells. Multiple studies indicate that its functional activity is crucial for the invasive characteristics of cancer cells, making it a promising target for therapy [4]. For instance, in breast cancer cells, the activation of TMEM16A promotes cell migration and invasion [79]. In vivo experiments have demonstrated that inhibiting the expression of TMEM16A effectively reduces cancer cell metastasis [67]. In lung cancer research, it has been found that TMEM16A is involved in the proliferation, migration, and invasion of cancer cells, with inhibition of its expression or activity significantly suppressing the growth and metastasis of lung cancer cells [65]. TMEM16A's involvement in numerous signaling pathways, particularly the activation of the MAPK pathway, is considered an important mechanism affecting tumor growth [2]. In prostate cancer, the overexpression of TMEM16A activates the MAPK pathway, promoting the proliferation and survival of cancer cells [65]. In colorectal cancer, studies have shown that Pt(IV) derivatives can effectively inhibit cancer cell metastasis by suppressing TMEM16A and related downstream signaling pathways [63]. In esophageal squamous cell carcinoma, TMEM16A interacts with EGFR to jointly regulate the metastasis and growth of cancer cells, with combined inhibition of these two targets demonstrating a synergistic anti-tumor effect [64].

Furthermore, in—depth research on the structure and function of TMEM16A has also provided new ideas for its application in cancer treatment. By analyzing the crystal structure of TMEM16A, researchers can design more precise inhibitors targeting this target [66]. Meanwhile, studies on the expression patterns and functional differences of TMEM16A in different cancer types can also contribute to the realization of precise cancer treatment, improving treatment efficacy and reducing side effects.

#### Future research directions

Given the increasing recognition of TMEM16A's role in cancer biology, future investigations should prioritize several essential domains. First, it is crucial to clarify the specific signaling pathways that TMEM16A utilizes to exert its tumor-promoting effects. Gaining a deeper understanding of its interactions with pathways such as MAPK could yield valuable insights regarding its involvement in malignant transformation and metastasis [4]. Second, the development of selective inhibitors aimed at TMEM16A signifies a potentially fruitful therapeutic pathway. For example, luteolin, a natural compound, has been identified as a powerful ANO1 inhibitor, showing significant anticancer properties by reducing ANO1 expression and activity in prostate cancer cells [5]. Further investigations into bioactive compounds might reveal additional therapeutic agents that effectively target TMEM16A. Lastly, investigating the potential of TMEM16A as a prognostic biomarker in cancer deserves attention. The correlation between TMEM16A expression levels and disease progression in certain cancers suggests it could serve as a useful instrument for patient stratification and treatment planning [11]. Future research should strive to confirm the efficacy of TMEM16A as a prognostic marker across multiple cancer types, thus augmenting its clinical relevance. In summary, continuous exploration of the function of TMEM16A in cancer biology, along with novel therapeutic approaches, offers significant promise for enhancing treatment outcomes in cancer, especially for prostate cancer and other malignancies linked to TMEM16A.

Developing TMEM16A-targeted therapies for clinical use will require validation of ANO1 as a prognostic biomarker in large-scale clinical cohorts across diverse cancer types by integrating its expression levels with patient survival outcomes and treatment responses, design of clinical trials to evaluate the safety and efficacy of TMEM16A inhibitors like luteolin or Etoposide as monotherapies or in combination with immunotherapeutic agents such as PD-1/PD-L1 blockers based on preclinical evidence of synergistic anti-tumor activity, and optimization of drug delivery systems to overcome barriers within the tumor microenvironment—such as developing nanocarrier-based formulations to enhance drug accumulation in tumor tissues and improve penetration into metastatic lesions—as illustrated in Fig. 3.

## Conclusion

The examination of TMEM16A has shed light on its vital function as a therapeutic target in prostate cancer, highlighting its importance in enhancing our comprehension of cancer biology. TMEM16A functions as a calcium-activated chloride channel (CaCC) that exhibits high expression levels in several prostate cancer cell lines, especially in metastatic instances such as LNCaP and PC-3 cells [15–25]. This heightened expression is associated with unfavorable prognosis and advanced clinical characteristics, as evidenced by correlations with TNM staging and Gleason scores observed in human prostate cancer specimens [1]. Moreover, ANO1 has been investigated in additional cancers, including head and neck squamous cell carcinoma (HNSCC), where its amplification and elevated expression were associated with poor survival outcomes, indicating its potential as a prognostic marker and therapeutic target [11]. Studies have shown that the silencing of TMEM16A through small hairpin RNAs (shRNAs) results in considerable decreases in cell proliferation, migration, and invasion within prostate cancer models. Importantly, the intratumoral administration of TMEM16A shRNA not only curtailed tumor growth but also enhanced survival rates in orthotopic nude mice [1]. Additionally, the discovery of specific inhibitors, such as luteolin, has demonstrated potential in downregulating TMEM16A activity, thus hindering cancer cell proliferation and migration. This points to a dual mode of action that includes both the inhibition of channel activity and a reduction in protein expression [5]. Furthermore, the overexpression of ANO1 has been shown to promote cell movement and invasion, reinforcing the notion that targeting this channel could diminish tumorigenic properties [4].

The relationship between TMEM16A and various signaling pathways, especially the MAPK pathway, has been a significant focus of contemporary research. An increase in TMEM16A expression has been associated with heightened activation of extracellular signal-regulated kinase (ERK), which in turn facilitates tumor growth and proliferation. The pharmacological blocking of this pathway mitigates the effects driven by TMEM16A, emphasizing the channel's involvement in cancer progression [2]. Taken together, these results highlight TMEM16A not only as a potential biomarker for prostate cancer but also as a promising target for therapeutic strategies, setting the stage for the advancement of targeted treatments that may greatly influence patient management and outcomes.

To summarize, the findings related to TMEM16A offer valuable insights into its role in the development of prostate cancer and underscore the potential benefits of focusing on this channel for treatment. The integration of molecular biology, pharmacology, and oncogenesis in investigating TMEM16A marks a notable progress in our comprehension of cancer processes, establishing TMEM16A as a noteworthy target for upcoming therapies in prostate cancer. Additional research into its function in various cancer types will deepen our knowledge and may result in new therapeutic approaches.

## Data Availability

No datasets were generated or analysed during the current study.
